# Supplementary material for: Analyzing Flow Cytometry or Targeted Gene Expression Data Influences Clinical Discoveries—Profiling Blood Samples of Pancreatic Ductal Adenocarcinoma Patients
Source: Cancers (Basel). 2023 Aug 31;15(17):4349. doi: 10.3390/cancers15174349 (PMC10486875; doi:10.3390/cancers15174349)
Supplement: Supplementary file 1 [file cancers-15-04349-s001.zip › Supplementary Figures.pdf]

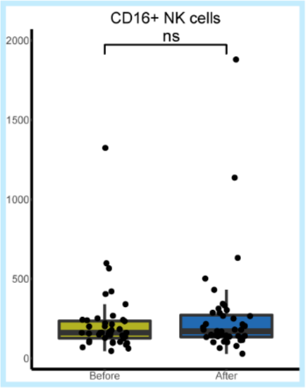

Supplementary Figure S1: The effect of one cycle FFX-Lipeg on the immune cells measured by flow cytometry. *The lymphocytes' (light blue) subtype CD16+ NK cells were not significantly altered by the treatment.*

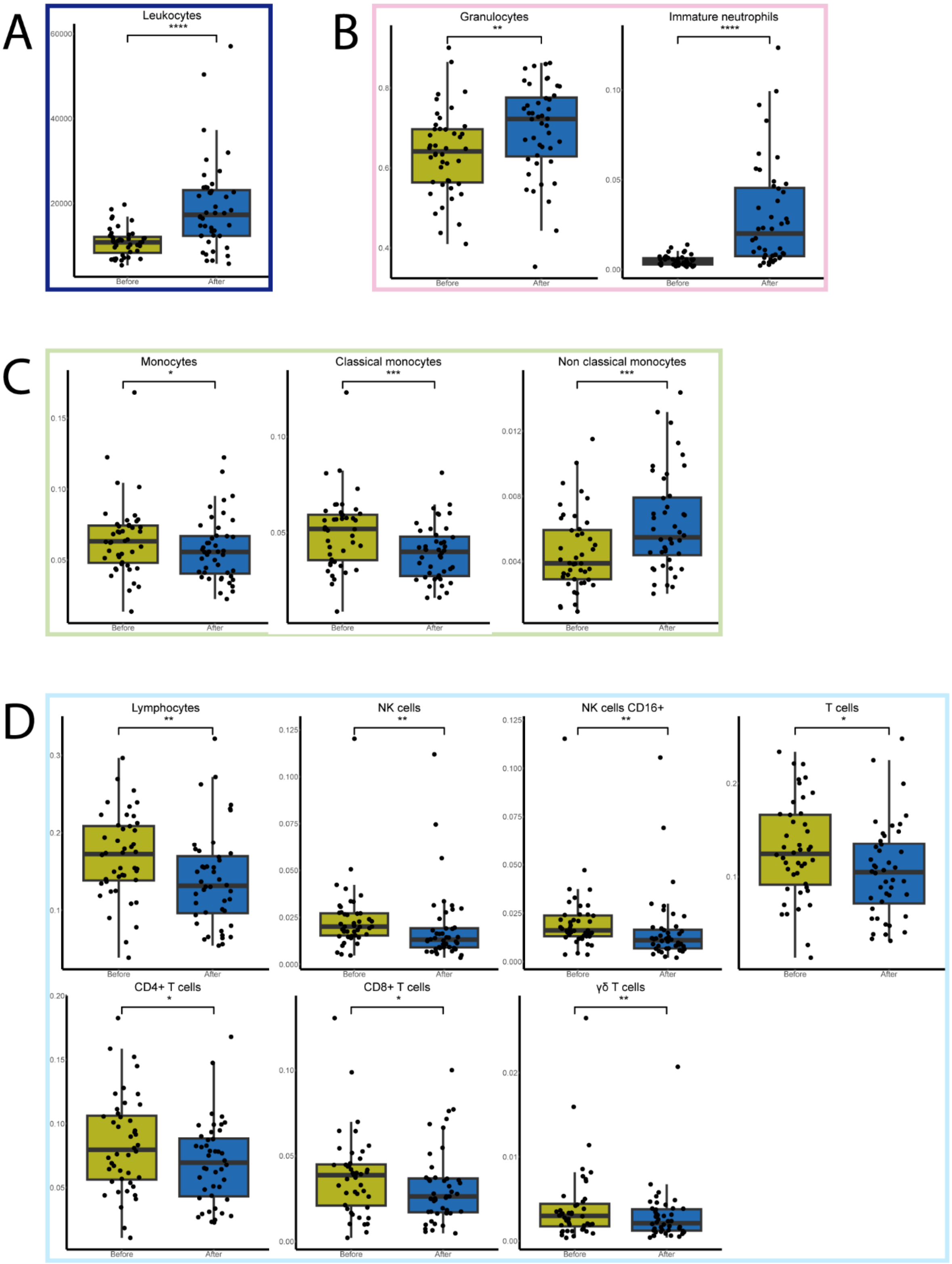

Supplementary Figure S2: The effect of one cycle FFX-Lipeg on the immune cells measured by flow cytometry relative to the total number of leukocytes. *(A) The total number of leukocytes. (B) The relative abundance of granulocytes (pink) including the subtypes that were significantly increased after treatment (blue) in comparison with before treatment (yellow). (C) The relative abundance of monocytes (green) including the subtypes that were significantly increased after treatment. (D) The relative abundance of lymphocytes (light blue) including the subtypes that were significantly increased after treatment.*

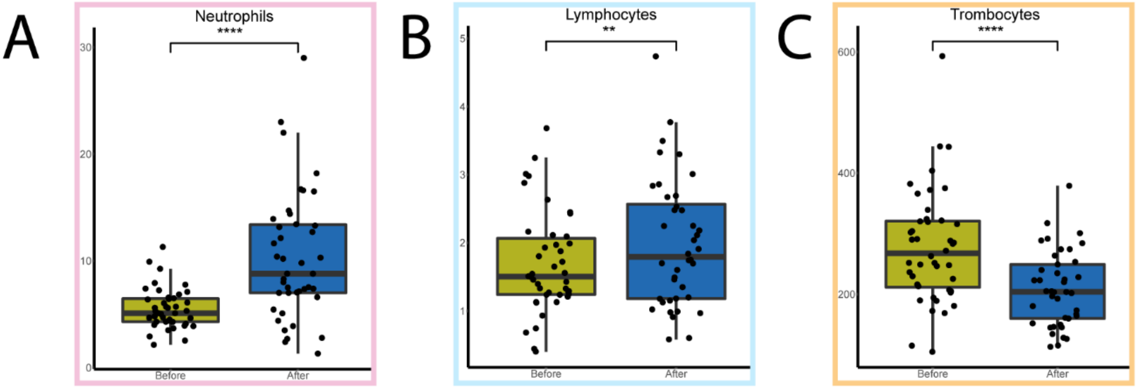

Supplementary Figure S3: The effect of one cycle FFX-Lipeg on the immune cells measured by complete blood count. (A) *The number of granulocytes' subtype neutrophils was significantly increased after treatment.* ***(B)*** *The number of lymphocytes significantly increased after treatment.* ***(C)*** *The number of thrombocytes significantly decreased after treatment.*

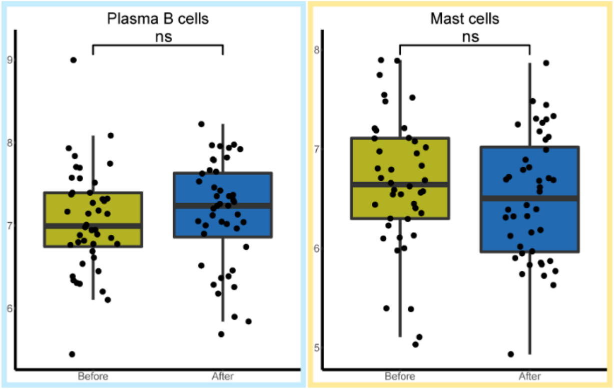

Supplementary Figure S4: The effect of one cycle FFX-Lipeg on the immune cells measured by targeted gene expression. *The lymphocytes' (light blue) subtype Plasma B cells and Mast cells (yellow) were not significantly altered by the treatment.*

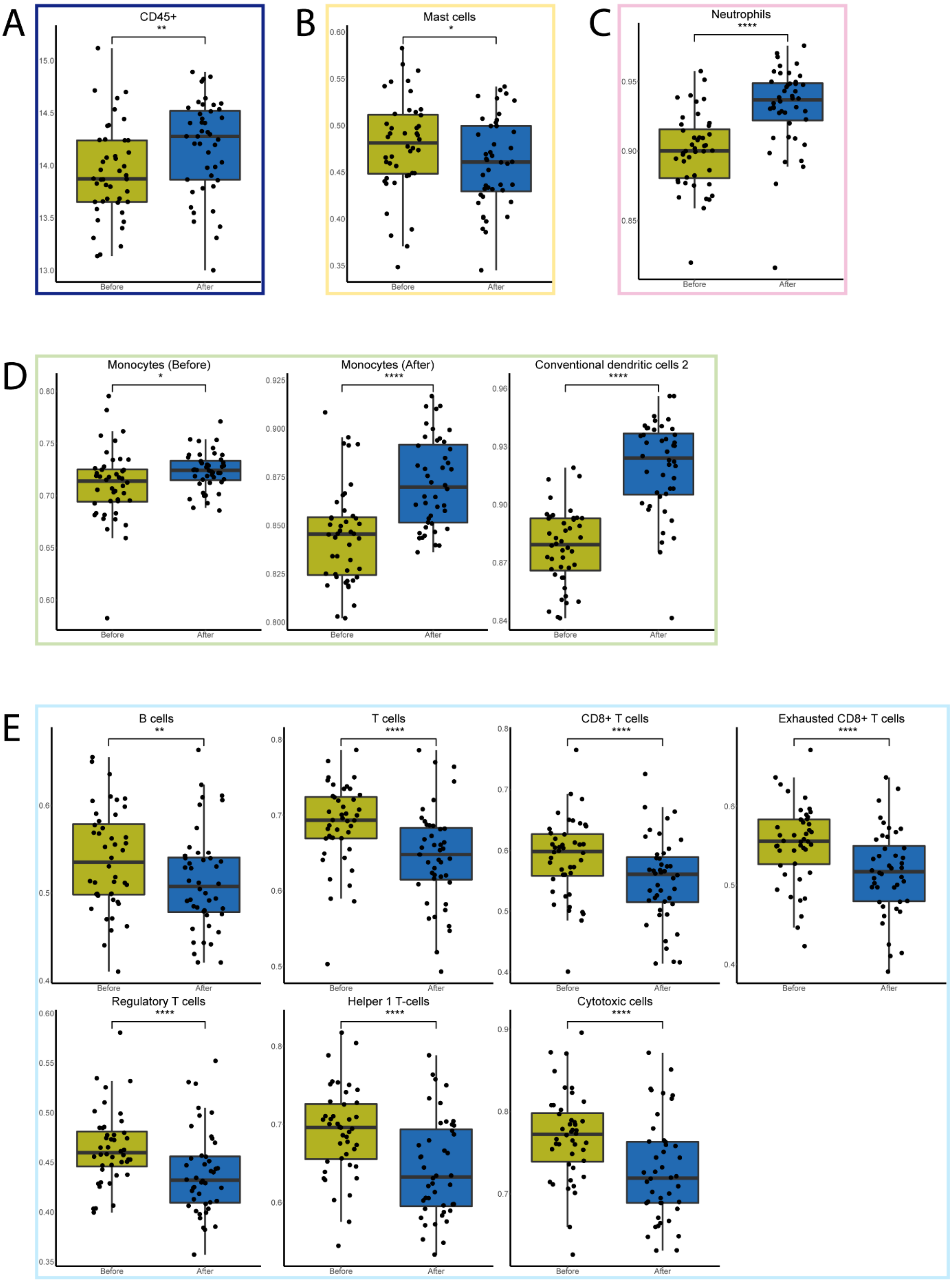

Supplementary Figure S5: The effect of one cycle FFX-Lipeg on the immune cells measured by targeted gene expression relative to the total CD45 expression. *(A) The total immune cell (dark blue) abundance was significantly increased after treatment (blue) in comparison with before treatment (yellow). (B) The relative abundance of mast cells (yellow) was significantly increased after treatment. (C) The relative abundance of granulocytes' (pink) subtype neutrophils was significantly increased after treatment. (D) The relative abundance of monocytes (green) including the subtypes was significantly increased after treatment. (DE) The relative abundance of lymphocytes (light blue) including the subtypes was significantly decreased after treatment.*

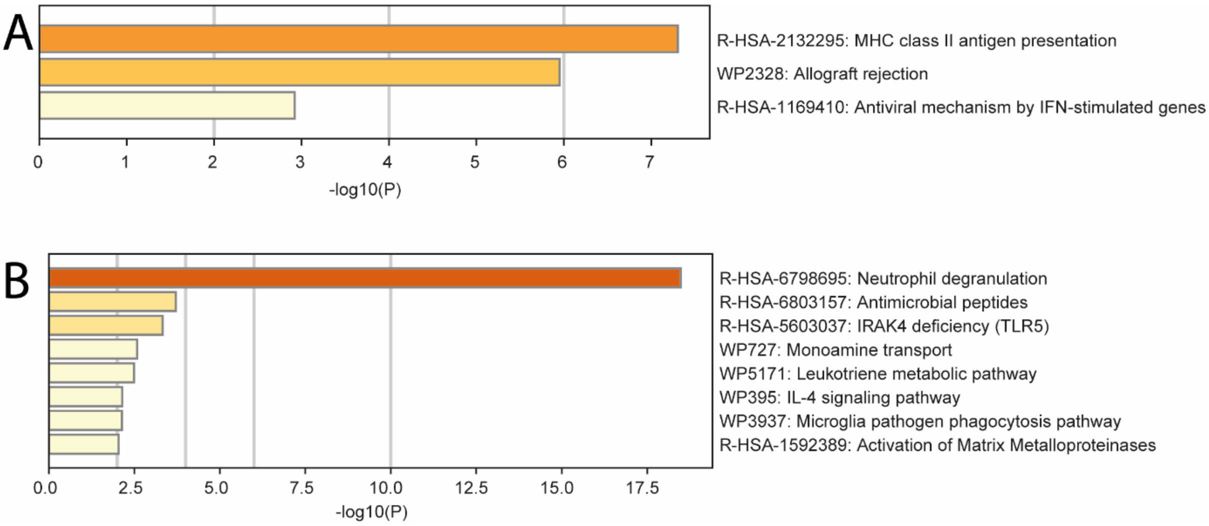

Supplementary Figure S6: Metascape enrichment analysis of the 39 downregulated and 115 upregulated genes after one FFX-Lipeg cycle. (A) *The 39 downregulated (P.BH ≤ 0.05 and log2 fold of change ≤ -0.5) genes showed that the FFX-Lipeg treatment negatively affected the MHC class II antigen presentation.* ***(B)*** *The 170 upregulated (P.BH ≤ 0.05 and log2 fold of change ≥ 0.5) genes showed an enriched neutrophil degranulation, IRAK4 deficiency (TLR5), leukotriene metabolic pathway, IL-4 signaling pathway, and activation of matrix metalloproteinases.*

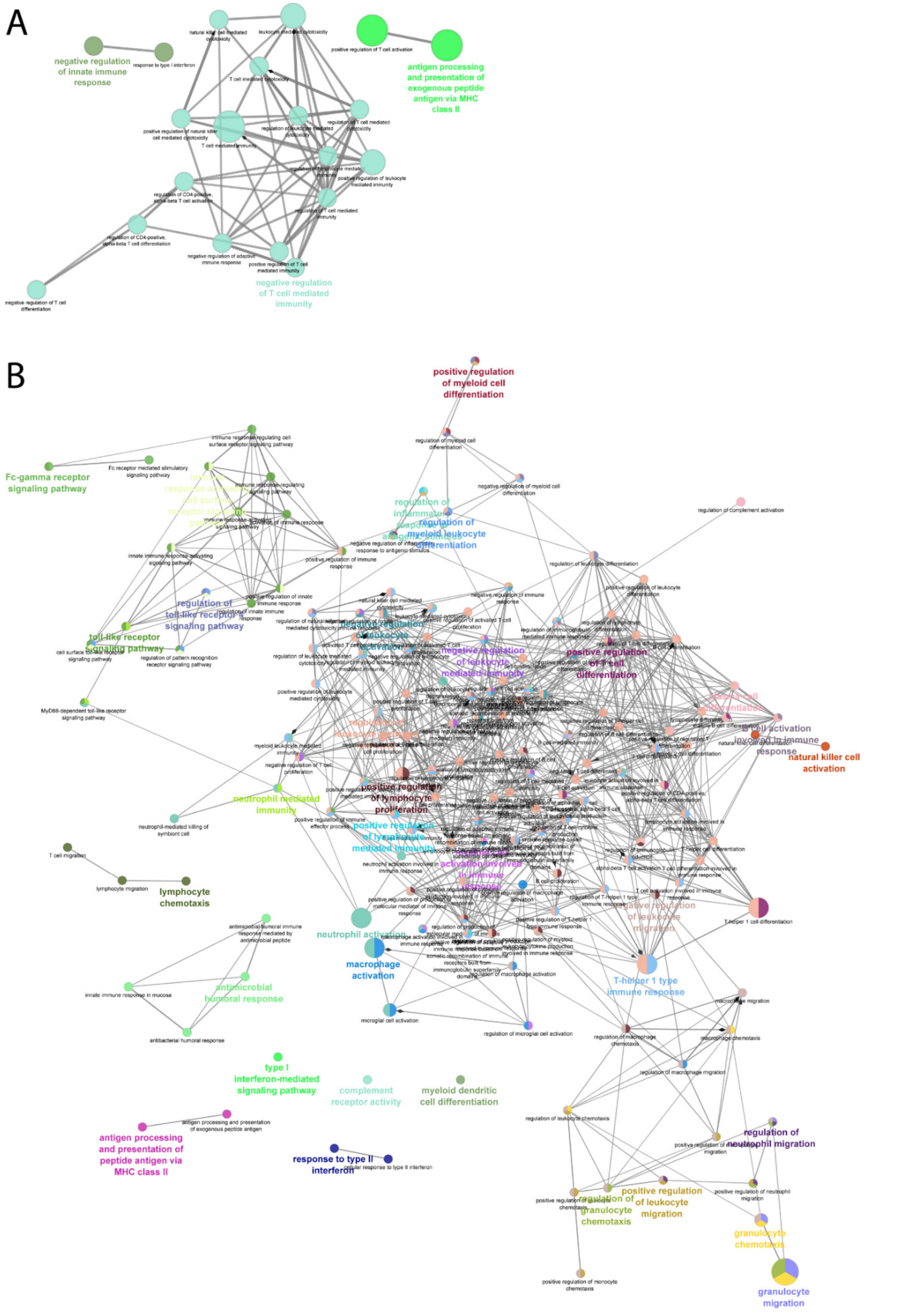

Supplementary Figure S7: ClueGo functional analysis of the 39 downregulated and 115 upregulated genes after one FFX-Lipeg cycle. (A) *The 39 downregulated (P.BH ≤ 0.05 and log2 fold of change ≤ -0.5) showed a negative regulation of T cell-mediated immunity and the innate immune response.* ***(B)*** *The 170 upregulated (P.BH ≤ 0.05 and log2 fold of change ≥ 0.5) genes showed enrichment of stimulated myeloid, and neutrophil cell-related functions and genes related to lymphocyte proliferation.*

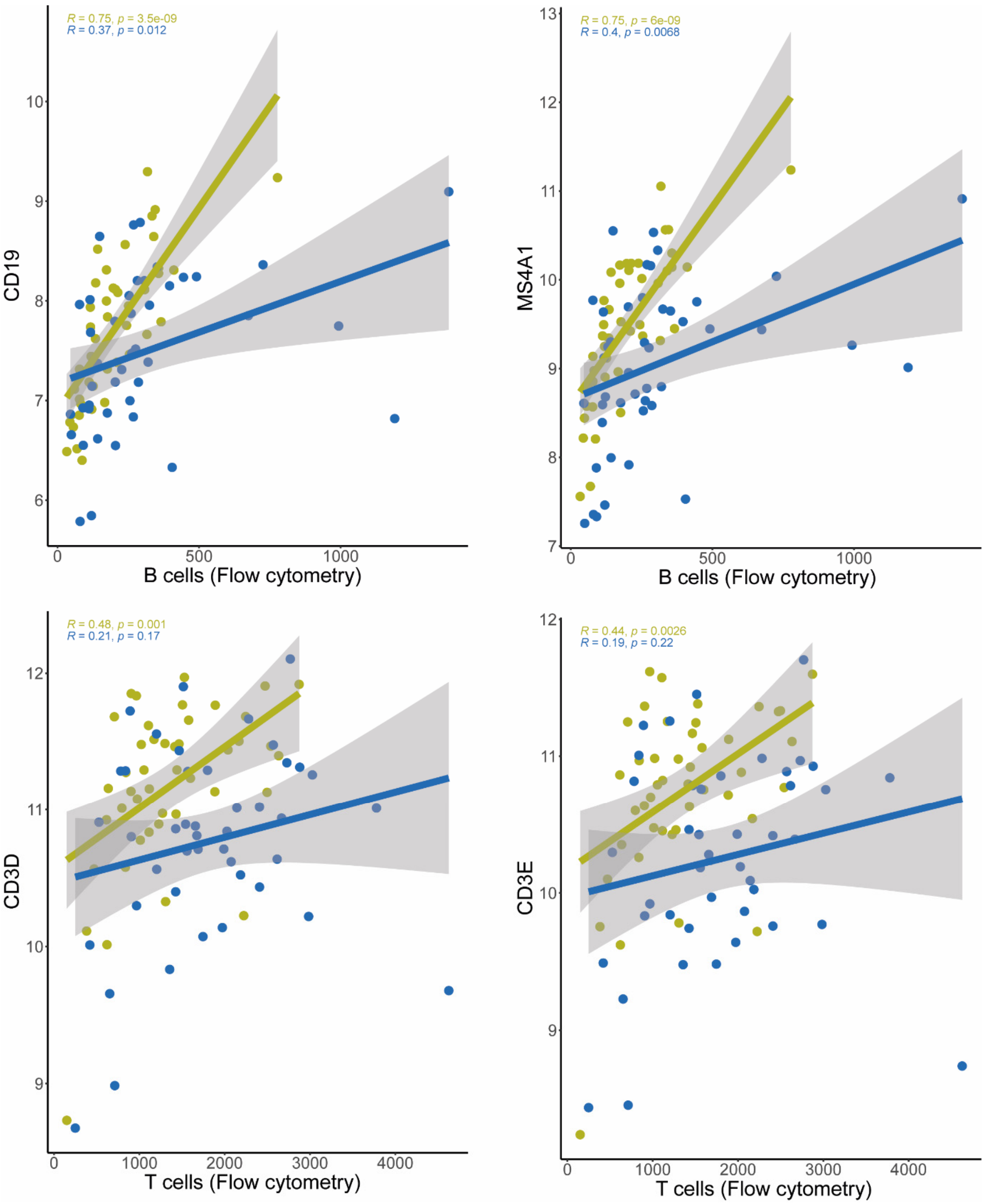

Supplementary Figure S8: Correlation analysis between the proteins targeted by flow cytometry with their corresponding marker genes in the targeted immune-gene expression profiling of the lymphocyte subtypes. *The correlation before FFX-Lipeg (yellow) is higher in comparison with the correlation after treatment.*
