# Supplementary material for: Analyzing Flow Cytometry or Targeted Gene Expression Data Influences Clinical Discoveries—Profiling Blood Samples of Pancreatic Ductal Adenocarcinoma Patients
Source: Cancers (Basel). 2023 Aug 31;15(17):4349. doi: 10.3390/cancers15174349 (PMC10486875; doi:10.3390/cancers15174349)
Supplement: Supplementary file 1 [file cancers-15-04349-s001.zip › Supplementary Table S1.pdf]

*Supplementary Table S1: Clinicopathological characteristics of patients included in the study.*

| | **ALL PATIENTS (N = 44)** |
|---|---|
| **AGE (Y), MEDIAN (RANGE)** | 64 (47 – 78) |
| **GENDER** | |
| FEMALE | 19 (43%) |
| MALE | 25 (57%) |
| **SMOKING STATUS** | |
| NEVER | 18 (41%) |
| CURRENT | 10 (23%) |
| FORMER | 16 (36%) |
| **ALCOHOL STATUS** | |
| NEVER | 18 (41%) |
| CURRENT | 20 (45%) |
| FORMER | 6 (14%) |
| **BASELINE CA19-9 (U/ML), MEDIAN (RANGE)** | 143 (0, 26500) |
| **BASELINE CEA (μG/L), MEDIAN (RANGE)** | 4.1 (0.7, 299.0) |
| **BASELINE SII, MEDIAN (RANGE)** | 866 (201, 7250) |
| **BASELINE NLR, MEDIAN (RANGE)** | 3.3 (1.3, 19.8) |
| **BASELINE BILIRUBIN (μMOL/L), MEDIAN (RANGE)** | 9.0 (3.0, 30.0) |
| **BASELINE CRP (MG/L), MEDIAN (RANGE)** | 4.9 (0.4, 91.0) |
| **DISEASE STAGE** | |
| (BORDERLINE) RESECTABLE | 14 (32%) |
| LAPC | 19 (43%) |
| METASTATIC DISEASE | 11 (25%) |
| **TOTAL CYCLES OF FOLFIRINOX, MEDIAN (RANGE)** | 8 (1, 12) |
| **PROGRESSION AFTER 4 CYCLES (RECIST 1.1)** | |
| STABLE DISEASE | 31 (71%) |
| PARTIAL RESPONSE | 8 (18%) |
| PROGRESSIVE DISEASE | 5 (11%) |
| **OS (M), MEDIAN (RANGE)** | 9 (1, 26) |
