# Supplementary material for: Analyzing Flow Cytometry or Targeted Gene Expression Data Influences Clinical Discoveries—Profiling Blood Samples of Pancreatic Ductal Adenocarcinoma Patients
Source: Cancers (Basel). 2023 Aug 31;15(17):4349. doi: 10.3390/cancers15174349 (PMC10486875; doi:10.3390/cancers15174349)
Supplement: Supplementary file 1 [file cancers-15-04349-s001.zip › Supplementary Table S2.pdf]

Supplementary Table S2: The definition of infiltrated immune cells was based on 41 genes that showed a pairwise similarity higher than 0.6 and defined thirteen different immune cell types. Genes depicted in bold are selected to define the immune cells.

Cell type	# Candidate genes	Mean pairwise similarity statistics in All samples	Mean pairwise similarity statistics in Before	Mean pairwise similarity statistics in After	Marker genes (bold are selected)
B-cells	*5*	*0.7*	*0.8*	*0.7*	***BLK, CD19, CD22, CR2, MS4A1***
Plasma B-cells	*4*	*CD38, TNFRSF17*<br>*0.6*	*CD38, TNFRSF17*<br>*0.6*	*CD38, TNFRSF17*<br>*0.5*	*CD27,* ***CD38****, CD38.1, SLAMF7,* ***TNFRSF17***
Regulatory B-cells		*0.1*	*-0.2*	*0.0*	*CD1D, CD5*
CD45	*1*				***PTPRC***
Cytotoxic cells	*7*	*0.7*	*0.7*	*0.7*	***GZMA, GZMB, GZMH, KLRB1, KLRD1, KLRK1,*** *KLRK1.1****, PRF1, NKG7***
Dendritic cells	*2*	*0.4*	*0.4*	*0.4*	*CD1A, CD1C*
Conventional Dendritic cells 1	*2*	*-0.4*	*-0.2*	*-0.4*	*BTLA, XCR1*
Conventional Dendritic cells 2	*2*	*0.8*	*0.8*	*0.8*	***ITGAM, ITGAX***
Macrophages	*2*	*-0.1*	*-0.1*	*-0.1*	*CD68, FCGR2A*
Antigen Presenting cells	*2*	*-0.1*	*0.0*	*0.0*	*CD80, CD80.1, CD86*
M2 Macrophages	*2*	*0.3*	*0.3*	*0.4*	*CD163, MRC1*
Mast cells	*2*	*MS4A2, CPA3, HDC*<br>*0.8*	*MS4A2, CPA3, HDC*<br>*0.8*	*MS4A2, CPA3, HDC*<br>*0.8*	***MS4A2****, MS4A2.1, TPSAB1,* ***CPA3****,* ***HDC***
Monocytes	*3*	***CD14, TLR2***<br>*0.5*	***CD14, CD33***<br>*0.5*	*CD14, TLR2*<br>*0.8*	*CD14, CD33, TLR2, TLR2.1*
Neutrophils	*2*	*0.7*	*0.7*	*0.7*	***CSF3R,*** *CSF3R.1****, FCGR3A,*** *FCGR3A.1****, FPR1, SIGLEC5, FCAR***
T-cells	*5*	*0.9*	*0.9*	*0.9*	***CD3D, CD3E, CD3G, CD6, SH2D1A***
CD4+ T-cells	*2*	*-0.4*	*-0.4*	*-0.2*	*CD4, SELL*
CD8+ T-cells	*2*	*0.8*	*0.9*	*0.8*	***CD8A, CD8B***
Exhausted CD8+ T-cells	*3*	*LAG3, TIGIT*<br>*0.6*	*LAG3, TIGIT*<br>*0.6*	*LAG3, TIGIT*<br>*0.6*	***LAG3****, PDCD1, PDCD1.1****, TIGIT***
Helper 1 T-cells	*1*				***TBX21***
Regulatory T-cells	*2*	*0.6*	*0.6*	*0.7*	***FOXP3, IL2RA***
