# Supplementary material for: Analyzing Flow Cytometry or Targeted Gene Expression Data Influences Clinical Discoveries—Profiling Blood Samples of Pancreatic Ductal Adenocarcinoma Patients
Source: Cancers (Basel). 2023 Aug 31;15(17):4349. doi: 10.3390/cancers15174349 (PMC10486875; doi:10.3390/cancers15174349)
Supplement: Supplementary file 1 [file cancers-15-04349-s001.zip › Supplementary Table S3.pdf]

Supplementary Table S3: The nSolver pathway definitions.

Pathway	Genes
B-cell Functions	CD274, CD79B, CD1D, CD3E, CTLA4, CD70, MS4A1, CD5, CD86, ADA, CXCR5, SOCS1, CD27, BLK, IRF4, PTPRC, CR2, CD19, FAS, CD80, TNFRSF14, CD38
NK Cell Functions	KLRK1, IL12RB1, ITGA1, IL18R1, KLRD1, KLRG1, CCR1, KLRB1, CXCR3, IL18RAP, CD2, CD7, KLRF1, KIR3DL1, NCR1, KLRC1, KLRC2, KIR_Activating_Subgroup_2, IFNG, IRF1, IL18, LILRB1, IL12RB2, KIR_Inhibiting_Subgroup_1, KIR_Inhibiting_Subgroup_2, KIR_Activating_Subgroup_1, IL12A
T-Cell Functions	IL4R, IL12RB1, ITGA1, IL18R1, CD274, STAT4, TBX21, CCR1, LCP1, MAF, CD8A, TIGIT, CXCR3, CCR4, IL18RAP, TP53, CD1D, CD2, CD3E, LCK, CTLA4, LAG3, CD70, CD7, CD1C, CD5, CD86, ADA, DPP4, CXCR5, CD8B, CD3G, STAT6, SOCS1, CCR5, CD27, IDO1, IRF4, PTPRC, IFNG, FAS, TNFSF14, IRF1, FOXP3, EOMES, IL18, CD80, EGR1, LILRB1, IL12RB2, TNFRSF14, CXCL10, CD47, CXCL9, IL13RA1, F2RL1, IL12A, CD38
Differentiation and Maintenance of Myeloid Cells	ERG, S100A9, CTTNBP2, S100A11, S100A4, GADD45B, MSC, TOP2A, CEBPD, MCM5, LAPTM5, MSH2, HES4, S100A10, ELL2, GATA2, CEBPA, PDZK1IP1, HIP1R, MARCO.1, CLIC4, CEBPG, PFDN6, KLF4, MAFF, ZFP92, GATA1, ID2, IKZF1, EGR3, CD38.1, CYTIP, NMB, MAFB, NKG7, FPR3, RUNX2, PBX1, PBX3
